# Supplementary material for: Ligand Modulation on the Various Structures of Three Zinc(II)-Based Coordination Polymers for Antibiotics Degradation
Source: Molecules. 2023 Mar 24;28(7):2933. doi: 10.3390/molecules28072933 (PMC10095641; doi:10.3390/molecules28072933)
Supplement: Supplementary file 1 [file molecules-28-02933-s001.zip › molecules-2270509-supplementary.pdf]

Supplementary Information

Ligand Modulation on the Various Structures of Three Zinc(II)-Based Coordination Polymers for Antibiotics Degradation

Min Xiong [1], Ying-Gui Xia [1], Lu Lu [1,*], Jun Wang [1], A. Mohanty [2,*], Yu Wu [1], Hiroshi Sakiyama [3], Mohd. Muddassir [4] and Ying Pan [5,*]

[1] School of Chemistry and Environmental Engineering, Sichuan University of Science & Engineering, 643000 Zigong, China
[2] Department of Chemistry, Berhampur University, Berhampur 760007, Odisha, India
[3] Department of Science, Faculty of Science, Yamagata University, 1-4-12 Kojirakawa, Yamagata 990-8560, Japan
[4] Department of Chemistry, College of Sciences, King Saud University, Riyadh 11451, Saudi Arabia
[5] The First Dongguan Affiliated Hospital, Guangdong Medical University, Dongguan 523808, China

X-ray Crystallography

The single crystal X-ray diffraction data for **1-3** was collected using Bruker SMART APEX diffractometer having graphite monochromated MoKα radiation (λ = 0.71073 Å) employing ω-scan technique. The structure was solved using direct method (SHLEXS-2014) and refined deploying full-matrix least-square procedure based on F^2 (Shelxl-2014). All hydrogen atoms were generated geometrically and refined isotropically using a riding model, while, all non-hydrogen atoms were refined with anisotropic displacement parameters. Crystallographic details and selected bond dimensions for **1-3** are presented in Tables S1 and S2, respectively. CCDC: 2190809-21908011.

Photocatalytic Method

The photocatalytic activities of **1-3** (40 mg) were evaluated by the degradation of antibiotic pollutants in the aqueous solution under a UV-400 type photochemical reactor having 100 W mercury lamp (mean wavelength 365 nm). The aqueous solution of 50 mL 20 mg/L antibiotics was mixed with 0.01 mmol photocatalyst. The suspension containing antibiotics and photocatalysts was magnetically for 30 min stirred in the dark till adsorption-desorption equilibrium was established. 3.5 mL sample is extracted at 5 min intervals using 3 mL pipettor and centrifuged to remove the residual catalyst for analysis by UV visible spectroscopy spectrophotometer at an absorption wavelength applied to monitor the photocatalytic degradation. In addition, the control experiment was also accomplished in the following reaction conditions: (1) without photocatalyst under UV irradiation; (2) with photocatalyst under UV irradiation in the presence of 2 mL tert-butanol (*t*-BuOH); (3) 2 mL benzoquinone (BQ) was used instead of t-BuOH; (4) 2 mL ammonium oxalate (AO) was used instead of TBA. The degradation efficiency of antibiotics is defined as follows:

$$\text{Degradation efficiency} = (C_0\text{-}C)/C_0 \times 100\%$$

Where C_0 (mg/L) is the initial concentration of dyes, and C (mg/L) is the concentration of dyes at reaction time, t (min).

Table S1. Crystallographic data and structure refinement details for 1-3.

Parameter	1	2	3
Formula	$C_{20}H_{13}N_5O_6Zn$	$C_{19}H_{13}N_5O_4Zn$	$C_{38}H_{26}N_{10}O_8Zn_2$
Formula weight	484.72	440.71	881.43
Crystal system	Triclinic	Orthorhombic	Triclinic
Space group	*P-1*	*P212121*	*P-1*
Crystal Color	Yellow	Yellow	Yellow
a, Å	10.0778(10)	10.6283(8)	9.3026(9)
b, Å	10.1487(11)	16.4180(12)	11.3396(11)
c, Å	11.7163(12)	43.034(3)	11.6215(11)
α, °	103.902(2)	90	85.270(2)
β, °	95.506(2)	90	66.9920(1)
γ, °	101.016(2)	90	71.823(2)
V, Å ^{3}	1129.0(2)	7509.2(9)	1071.00(18)
Z	2	16	1
ρ_{calcd}, g/cm^3	1.423	1.559	1.367
μ, mm^{-1}	1.132	1.345	1.179
F(000)	490	3584	448
θ Range, deg	1.8-27.7	0.9-27.6	1.9-27.6
Reflection Collected	6909	45670	6490
Independent reflections (R_{int})	0.017	0.056	0.015
Reflections with $I > 2\sigma(I)$	4135	11907	4046
Number of parameters	289	1045	262
R_1, wR_2 ($I > 2\sigma(I)$)*	0.0390, 0.0976	0.0571, 0.1294	0.0332, 0.0886
R_1, wR_2 (all data)**	0.0503, 0.1085	0.0937, 0.1450	0.0399, 0.0922

Table S2. Selected bond distances (Å) and angles (deg) for **1-3.**

		**1**	
Zn(1)-O(1)	1.971(2)	Zn(1)-N(1)	1.991(2)
Zn(1)-N(5)#1	2.017(2)	Zn(1)-O(5)#2	2.016(2)
Zn(1)-O(6)#2	2.603(2)		
		**2**	
Zn(1)-O(1)	1.972(5)	Zn(1)-N(1)	2.060(7)
Zn(1)-N(6)	2.018(7)	Zn(1)-O(7)#1	2.017(6)
Zn(1)-O(8)#1	2.579(7)	Zn(2)-O(3)	1.984(5)
Zn(2)-O(5)	1.962(5)	Zn(2)-N(11)	1.998(6)
Zn(2)-N(10)#5	2.037(7)	Zn(3)-O(9)	1.980(5)
Zn(3)-O(10)	2.649(6)	Zn(3)-N(15)	2.023(7)
Zn(3)-N(16)	2.042(5)	Zn(3)-O(11)#2	2.203(8)
Zn(3)-O(12)#2	2.371(8)	Zn(4)-O(13)	1.966(5)
Zn(4)-N(20)	2.027(7)	Zn(4)-O(15)#3	2.062(6)
Zn(4)-O(16)#3	2.382(8)	Zn(4)-N(5)#4	2.051(7)
		**3**	
Zn(1)-O(1)	1.984(2)	Zn(1)-O(2)	2.664(2)
Zn(1)-O(3)	1.9556(18)	Zn(1)-N(1)	2.0126(18)
Zn(1)-N(5)#1	2.0324(18)		
		**1**	
O(1)-Zn(1)-N(1)	111.15(9)	O(1)-Zn(1)-N(5)#1	107.05(9)
O(1)-Zn(1)-O(5)#2	97.27(8)	O(1)-Zn(1)-O(6)#2	152.39(8)
O(1)-Zn(1)-N(5)#1	117.36(9)	O(5)#3-Zn(1)-N(1)	113.96(8)
O(6)#3-Zn(1)-N(1)	84.06(8)	O(5)#3-Zn(1)-N(5)#1	108.01(8)
O(6)#3-Zn(1)-N(5)#1	84.05(8)	O(5)#3-Zn(1)-O(6)#2	55.12(7)
		**2**	
O(1)-Zn(1)-N(1)	95.7(3)	O(1)-Zn(1)-N(6)	105.7(3)
O(1)-Zn(1)-O(7)#1	105.4(2)	O(1)-Zn(1)-O(8)#1	159.9(2)
N(1)-Zn(1)-N(6)	99.4(3)	O(7)#1-Zn(1)-N(1)	101.4(3)
O(8)#1-Zn(1)-N(1)	94.4(3)	O(7)#1-Zn(1)-N(6)	140.3(3)
O(8)#1-Zn(1)-N(6)	89.8(2)	O(7)#1-Zn(1)-O(8)#1	55.4(2)
O(3)-Zn(2)-O(5)	111.4(2)	O(3)-Zn(2)-N(11)	111.9(2)
O(3)-Zn(2)-N(10)#5	95.8(2)	O(5)-Zn(2)-N(11)	122.9(2)
O(5)-Zn(2)-N(10)#5	99.5(2)	N(10)#5-Zn(2)-N(11)	111.1(3)
O(9)-Zn(3)-O(10)	54.0(2)	O(9)-Zn(3)-N(15)	125.5(3)
O(9)-Zn(3)-N(16)	103.9(3)	O(9)-Zn(3)-O(11)#2	118.9(3)
O(9)-Zn(3)-O(12)#2	96.0(3)	O(10)-Zn(3)-N(15)	80.8(2)
O(10)-Zn(3)-N(16)	85.7(2)	O(10)-Zn(3)-O(11)#2	169.3(3)
O(10)-Zn(3)-O(12)#2	129.8(3)	N(10)-Zn(3)-N(16)	100.8(2)
O(11)#2-Zn(3)-N(15)	109.4(3)	O(12)#2-Zn(3)-N(15)	91.1(3)
		**3**	
O(1)-Zn(1)-O(2)	53.10(9)	O(1)-Zn(1)-O(3)	103.23(8)
O(1)-Zn(1)-N(1)	124.11(8)	O(1)-Zn(1)-N(5)#1	95.08(8)
O(2)-Zn(1)-O(3)	93.27(7)	O(2)-Zn(1)-N(1)	83.38(7)
O(2)-Zn(1)-N(5)#1	145.03(9)	O(3)-Zn(1)-N(3)	113.62(8)
O(3)-Zn(1)-N(5)#1	109.83(7)	N(1)-Zn(1)-N(5)#1	109.13(7)

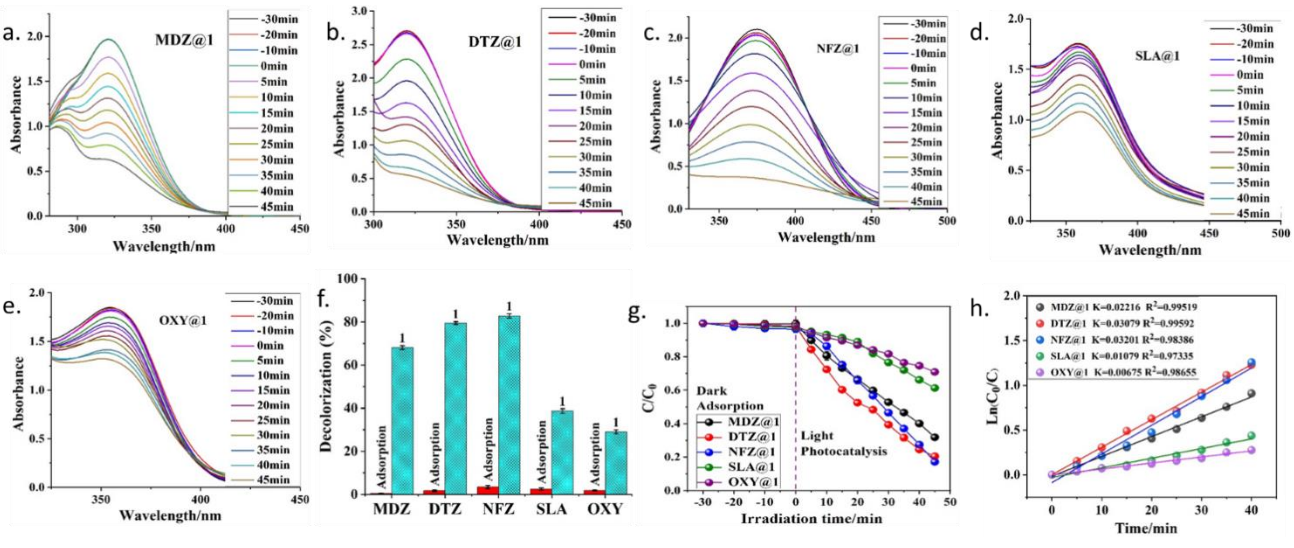

Figure S1. (**a**)–(**e**) MDZ, DTZ,NFZ, SLA and OXY degradation performances using **1**; (**f**) the comparative photocatalytic efficiency (**g**) the concentration changes of the antibiotics within 45 min in the presence of **1**; (**h**) pseudo first-order kinetics of all antibiotics degradation by **1**.

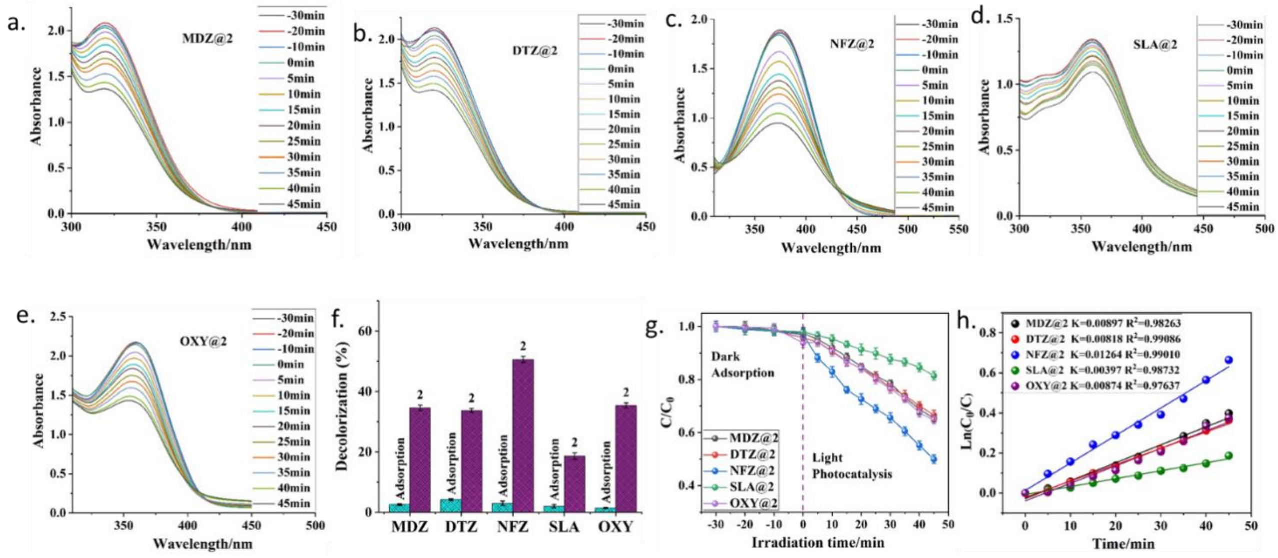

Figure S2. (a)–(**e**) MDZ, DTZ,NFZ, SLA and OXY degradation performances using **2**; (**f**) the comparative photocatalytic efficiency (**g**) the concentration changes of the antibiotics within 45 min in the presence of **2**; (**h**) pseudo first-order kinetics of all antibiotics degradation by **2**.

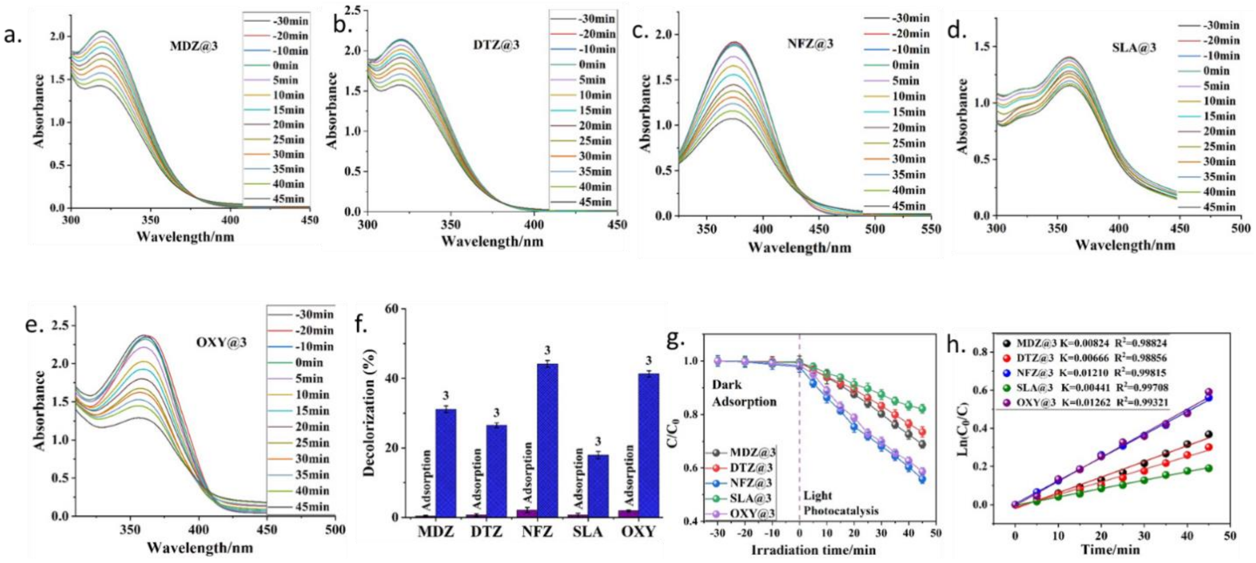

Figure S3. (**a**)–(**e**) MDZ, DTZ,NFZ, SLA and OXY degradation performances using **3**; (**f**) the comparative photocatalytic efficiency (**g**) the concentration changes of the antibiotics within 45 min in the presence of **3**; (**h**) pseudo first-order kinetics of all antibiotics degradation by **3**.

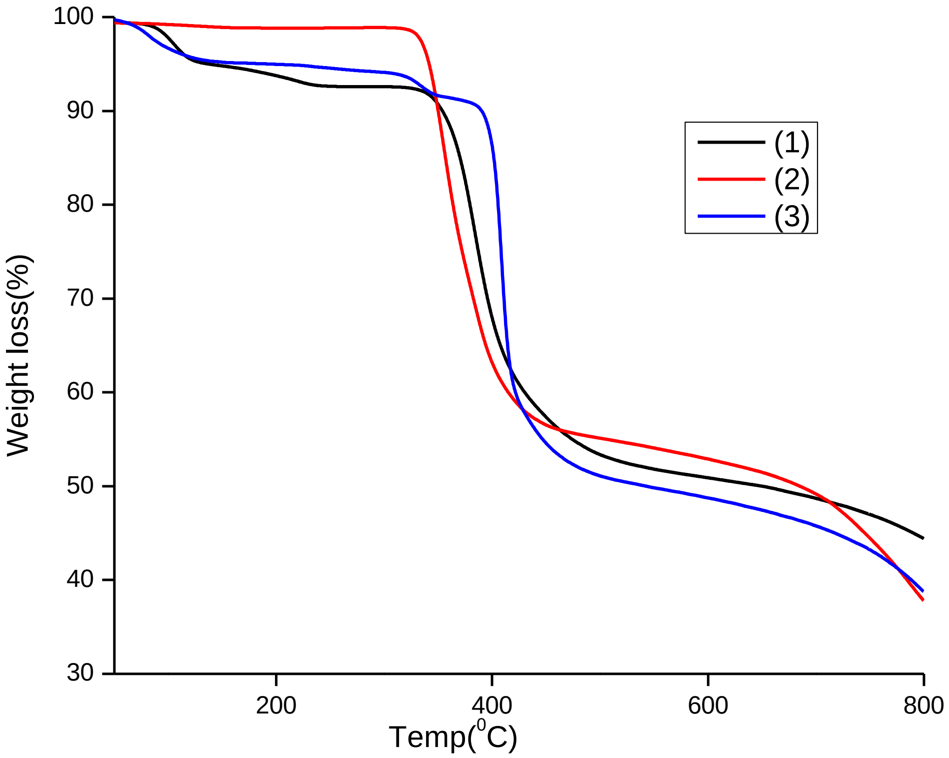

Figure S4. TGA of **1-3**.

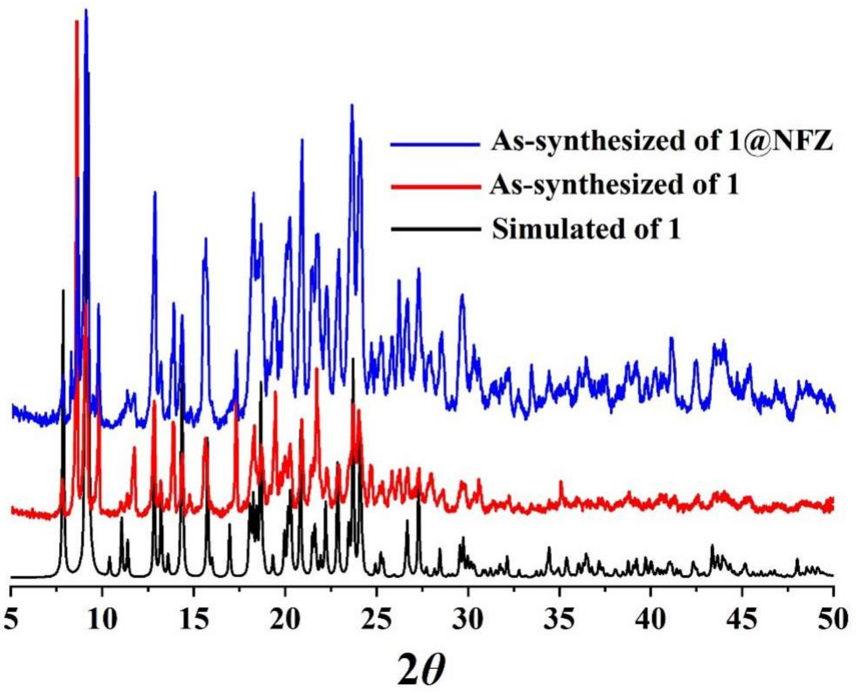

Figure S5. PXRD of **1.**

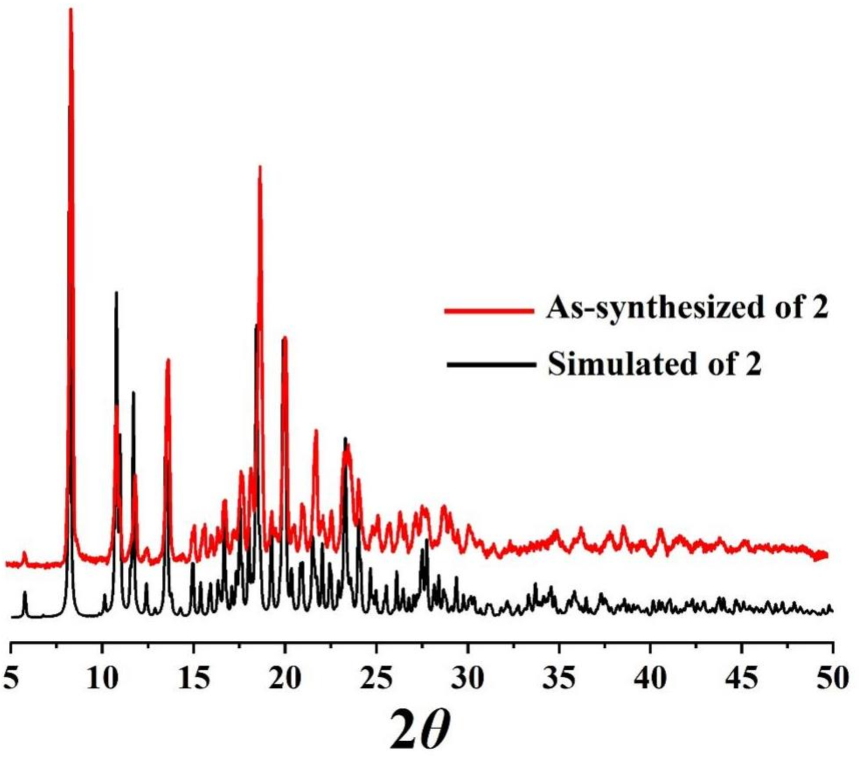

Figure S6. PXRD of **2.**

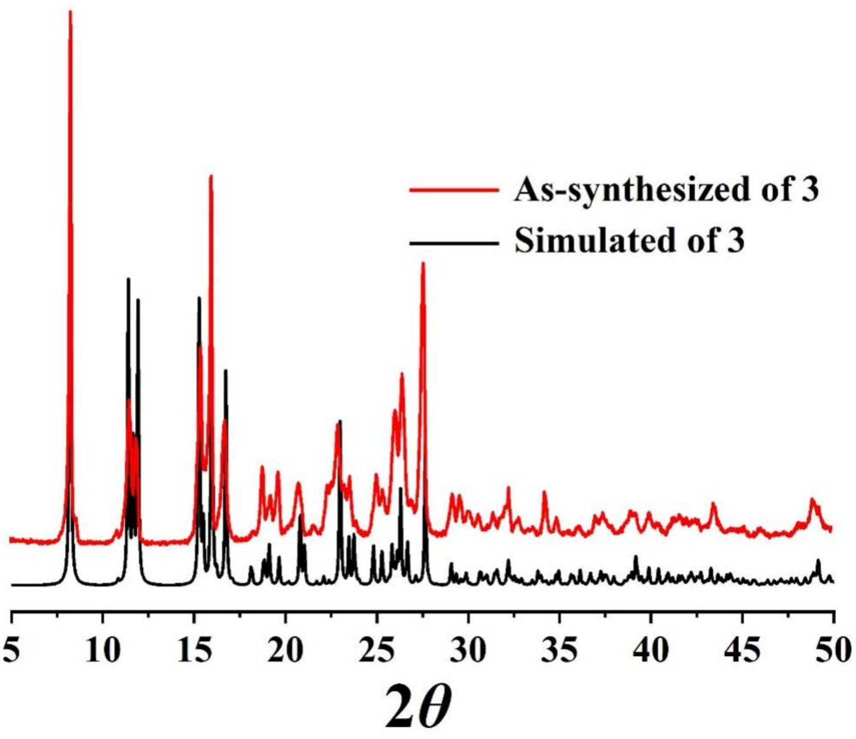

Figure S7. PXRD of **3.**

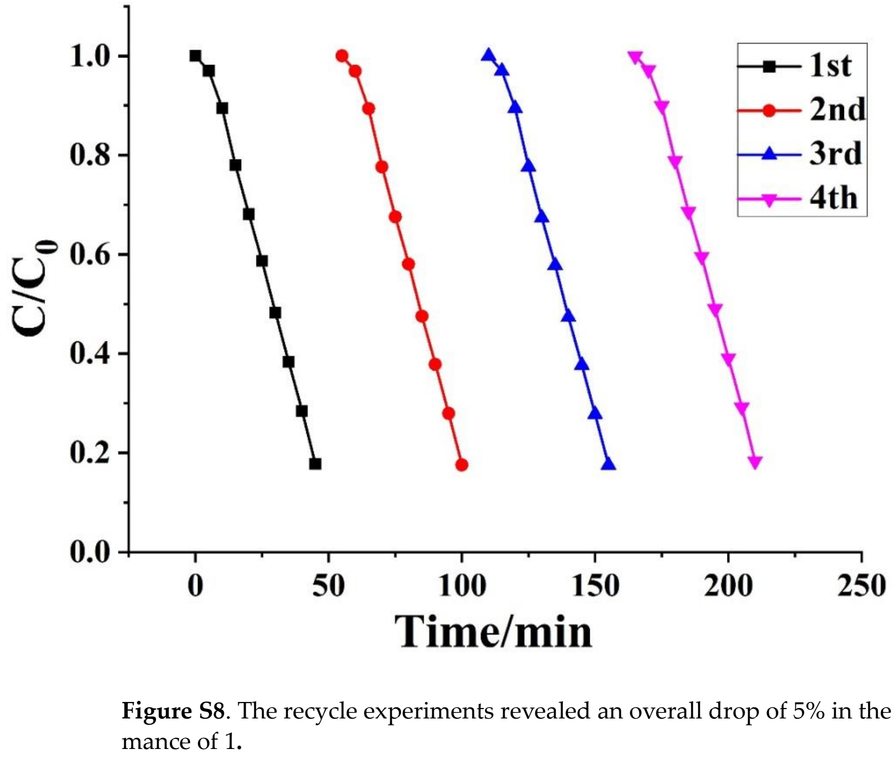

Figure S8. The recycle experiments revealed an overall drop of 5% in the photocatalytic performance of 1**.**

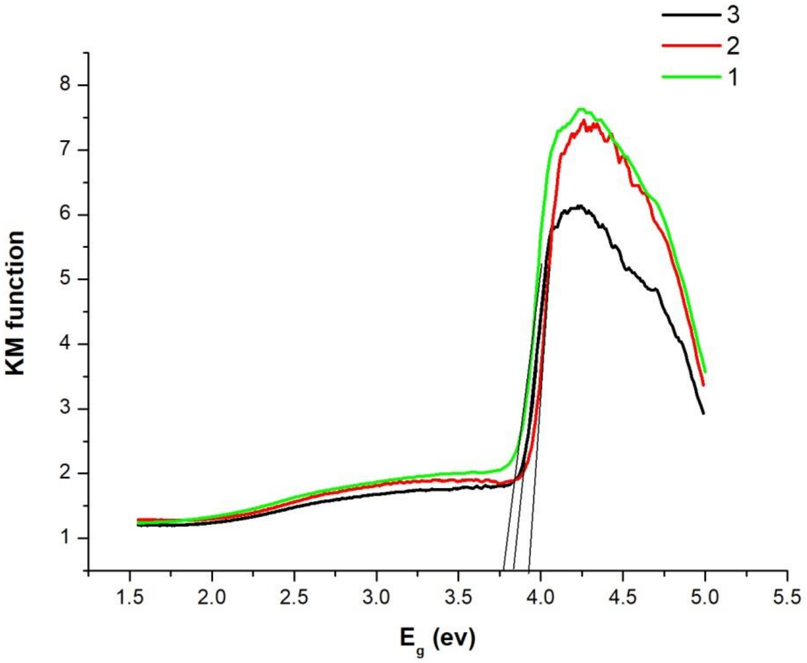

Figure S9. The diffuse reflectance (DR) UV-vis of **1-3.**
